# Silver and Gold NMR

**DOI:** 10.1155/MBD.1999.239

**Published:** 1999

**Authors:** Klaus Zangger, lan M. Armitage

**Affiliations:** Department of Biochemistry Molecular Biology and Biophysics, Medical School University of Minnesota, 5-130 BSBE, Building 312 Chumh Street SE Minneapolis MN 55455 USA

## Abstract

Silver and gold, together with copper, form the transition metal group IB elements in the periodic table and possess very different nuclear magnetic resonance (NMR) spectroscopic properties. While there is only one gold isotope (^197^Au), which has a spin of 3/2 and therefore a quadrupole moment, silver occurs in two isotopic forms (^109^Ag and ^109^Au), both of which have a spin 1/2 and similar NMR spectroscopic properties. The unfavorable properties of gold have prevented its NMR spectroscopic investigation thus far. On the other hand, there are several reports of silver NMR. However, the low sensitivity of silver, combined with its long relaxation times have rendered the direct detection of silver possible only with concentrations greater than a few tenth molar. Reviewed here are the general limitations of silver NMR and some techniques to partially overcome these limitations, as well as a summary of currently available chemical shift and scalar coupling data on ^109^Ag.


### SILVER AND GOLD NMR

Klaus Zangger and Ian M. Armitage*

Department of Biochemistry, Molecular Biology and Biophysics, Medical School,
University of Minnesota, 5-130 BSBE Building,
312 Church Street SE, Minneapolis, MN 55455, USA

Silver and gold, together with copper, form the transition metal group IB elements in the periodic table and possess very different nuclear magnetic resonance (NMR) spectroscopic properties. While there is only one gold isotope ($^{197}Au$), which has a spin of 3/2 and therefore a quadrupole moment, silver occurs in two isotopic forms ($^{107}Ag$ and $^{109}Ag$), both of which have a spin ½ and similar NMR spectroscopic properties. The unfavorable properties of gold have prevented its NMR spectroscopic investigation thus far. On the other hand, there are several reports of silver NMR. However, the low sensitivity of silver, combined with its long relaxation times have rendered the direct detection of silver possible only with concentrations greater than a few tenth molar. Reviewed here are the general limitations of silver NMR and some techniques to partially overcome these limitations, as well as a summary of currently available chemical shift and scalar coupling data on $^{109}Ag$.

### INTRODUCTION

Gold has a single isotope, $^{197}Au$, which has a nuclear spin of 3/2 and therefore a quadrupole moment. As a result of fast quadrupole relaxation, the resonances are extremely broad and weak. Due to the low NMR receptivity of $^{197}Au$ combined with the fast relaxation, no NMR studies of gold have been described in the literature. NMR spectroscopic investigations of gold compounds have, however, been reported where one has measured the NMR properties of other nuclei such as, $^{1}H$, $^{13}C$ or $^{31}P$, contained in the Au complex[1-3].

On the other hand, silver occurs in two isotopic forms $^{107}Ag$ and $^{109}Ag$, both of which have a spin of ½ and are therefore NMR receptive. Although $^{107}Ag$ has a slightly higher natural abundance (51.8% versus 48.2% for $^{109}Ag$), $^{109}Ag$ has usually been used for NMR studies due to its higher gyromagnetic ratio ($\gamma(^{109}Ag)/\gamma(^{107}Ag)=1.15$).

### GENERAL LIMITATIONS

The biggest inherent problem when studying silver by NMR spectroscopy is the low sensitivity, which for $^{109}Ag$ is just $1.01*10^{-4}$ when compared with an equal number of protons. One way to increase the sensitivity of low γ-nuclei is the use of the nuclear Overhauser effect (NOE) to enhance the signal of the insensitive nucleus by saturating a dipolar coupled, more sensitive partner. In the case of $^{109}Ag$ dipolar coupled to protons, this would give for the extreme narrowing limit a theoretical maximum enhancement of -10.7 ($(\gamma(^{1}H)/\gamma(^{109}Ag))/2$) if the relaxation would be entirely *via* the dipolar mechanism. The minus sign reflects the fact that $^{109}Ag$ has a negative gyromagnetic ratio. However, since the relaxation for $^{109}Ag$ is mainly caused by chemical shift anisotropy (CSA), only a small portion of the maximum enhancement is found in practical applications and the resulting NOE is often close to –1, which leads to a diminution or even cancellation of the silver signal[4]. In addition to the low gyromagnetic ratio, the unusually long relaxation time of silver spins makes its direct detection difficult. The spin-lattice or $T_1$ relaxation time has been measured to be in excess of several 100 seconds[5-7]. For $^{109}Ag$ in aqueous solutions of $AgNO_3$, it is between 900 and 1000s. The dipolar relaxation mechanism, which is almost exclusively responsible for the relaxation of protons, is not efficient for $^{109}Ag$ due to the lack of nearby protons. Instead, relaxation due to the chemical shift anisotropy mechanism is the most important contributor to relaxation of silver in asymmetric complexes and spin rotation in symmetric compounds[8]. To reduce the relaxation times to a practical range, one could add paramagnetic compounds, such as chromium, cobalt or iron compounds. It has been shown by Burges et al.[9], however, that the use of $Fe(NO_3)_3$ in this context induces significant chemical shifts.

Due to all the above-mentioned disadvantages, the direct detection of silver is limited to highly concentrated samples in the molar range and long acquisition times are unavoidable. To circumvent this problem, Brevard et al. have proposed to detect the silver resonance after transfer of magnetization from a scalar-coupled proton. The INEPT (Insensitive Nuclei Enhancement by Polarization Transfer)[10] experiment has been used by them for this purpose. Now the relaxation time of the proton is the determining factor for the duty cycle, which significantly reduces the experimental time and provides a gain in sensitivity of $\gamma(^1H)/\gamma(^{109}Ag)$. Other polarization transfer methods, like DEFT (Driven Equilibrium Fourier Transform)[11] and DEPT (Distortionless Enhancement by Polarization Transfer)[12] have also been reported. In order for these techniques to be useful, the $^{109}Ag$ nucleus must be coupled to a proton or another magnetic nucleus. The heteronuclear frequencies can also be observed indirectly when the magnetization at the silver spin is transferred to a proton or another nucleus for detection, leading to a gain of $(\gamma(Obs)/\gamma(^{109}Ag))^{3/2}$ where $\gamma(Obs)$ is the gyromagnetic ratio of the observed nucleus. Proton excitation and detection, as in a HSQC (Heteronuclear Single Quantum Coherence)[13] or HMQC (Heteronuclear Multiple Quantum Coherence)[14] experiment, gives the highest gain in signal to noise, as shown in Table 1.

**Table I.** Relative theoretical sensitivity for different ways of detecting $^{109}Ag$. If a nucleus other than $^{109}Ag$ is detected, the information about the $^{109}Ag$ nucleus has to be transmitted during the $t_1$ time of a multi-dimensional experiment.

| excited nucleus: | detected nucleus: | relative sensitivity: |
|---|---|---|
| $^{109}Ag$ | $^{109}Ag$ | 1 |
| $^{1}H$ | $^{109}Ag$ | 21.5 |
| $^{1}H$ | $^{1}H$ | 2143.4 |
| $^{13}C$ | $^{109}Ag$ | 5.4 |
| $^{13}C$ | $^{13}C$ | 67.8 |
| $^{15}N$ | $^{109}Ag$ | 2.2 |
| $^{15}N$ | $^{15}N$ | 7.2 |
| $^{31}P$ | $^{109}Ag$ | 8.7 |
| $^{31}P$ | $^{31}P$ | 223.3 |
| $^{19}F$ | $^{109}Ag$ | 20.2 |
| $^{19}F$ | $^{19}F$ | 1833.9 |

As becomes immediately obvious, the experimental time can be greatly reduced by observing a nucleus scalar-coupled to $^{109}Ag$. Furthermore, as mentioned earlier, the faster relaxation times of those nuclei coupled to $^{109}Ag$ reduces the necessary experimental time even further. The indirect detection of $^{109}Ag$ via proton[4], phosphorus[15,16] and fluourine[17] has been described.

### A BIOCHEMICAL APPLICATION OF $^{109}Ag$ NMR

The first indirect detected spectrum of $^{109}Ag$ in a biological system was obtained by S.S. Narula et al.[18]. The $^{1}H$-$^{109}Ag$ HMQC experiment was used to establish the metal to cysteine connectivities in the silver-substituted copper yeast metallothionein protein. Metallothioneins are small, cysteine-rich proteins that bind the essential metals Cu(I) and Zn(II) in two clusters. Based upon the properties of thermodynamic stability combined with high kinetic lability, MTs are thought to function biologically as intracellular distributors and mediators of metal-ions. Metallothioneins lack regular secondary structural elements, and the conformation is mainly determined by the metal to cysteine connectivities. Establishing these connectivities is, therefore, essential for a 3-dimensional structural analysis by NMR. However, the quadrupole moment of Cu(I) spins results in extremely fast relaxation and poor NMR receptivity. Substitution of Cu(I) with the isomorphic spin ½ $^{109}Ag$ nucleus overcomes this problem and provides a NMR sensitive probe. Narula etal. have used the $^{1}H$-$^{109}Ag$ HMQC experiment (Figure 1) to establish the connectivities of all seven silver atoms bound in yeast metallothionein to their respective cysteines. These connectivities are shown diagrammatically with the primary sequence in Figure 2.

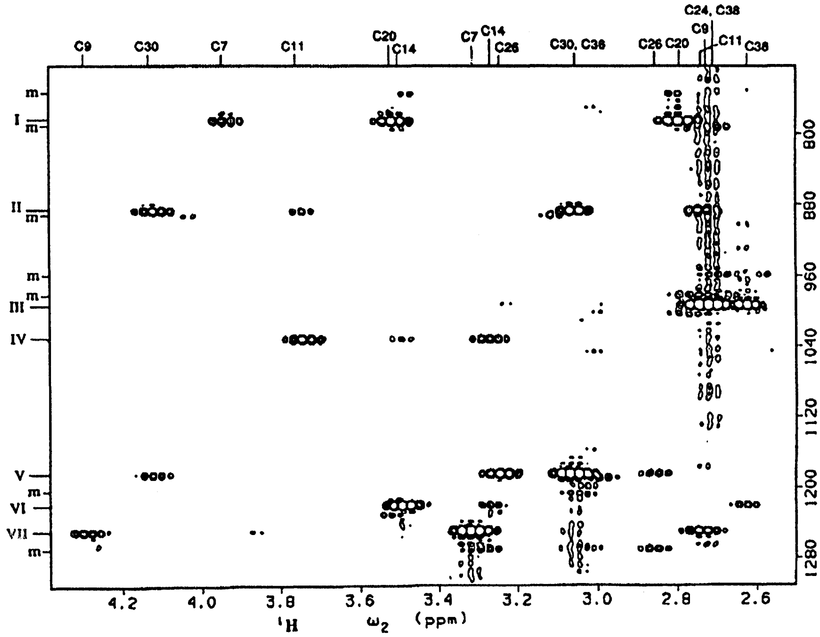

**Figure 1.**
$^{1}H$-$^{109}Ag$ HMQC showing the connectivities of all seven silver atoms with the cysteine β-protons in yeast metallothionein-I.

To this day, this study remains the only application of $^{109}Ag$ NMR to a biochemical problem.

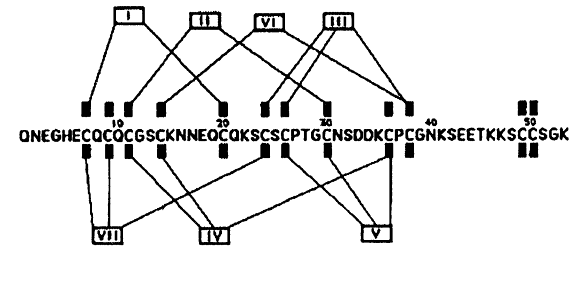

**Figure 2.**
Amino-acid sequence and Cys-Ag-Cys connectivities in yeast metallothionein-I.

### CHEMICAL SHIFTS

The chemical shift range reported for $^{109}Ag$ (Figure 3) extends from –250ppm to +2519ppm[17,19], using a 1M solution of $Ag(NO_3)$ as reference at 0ppm. The silver resonance frequency is sensitive to the oxidation state, with $Ag^{III}$ having shifts further upfield (beyond 2000ppm), the nature of the ligand and the coordination number. The latter increases according to:

$AgL_2 < AgL_3 < AgL_4$

Further, the nature of the solvent and temperature also contribute to the measured chemical shift. A depiction of the chemical shift scale for different ligands is shown in Figure 3.

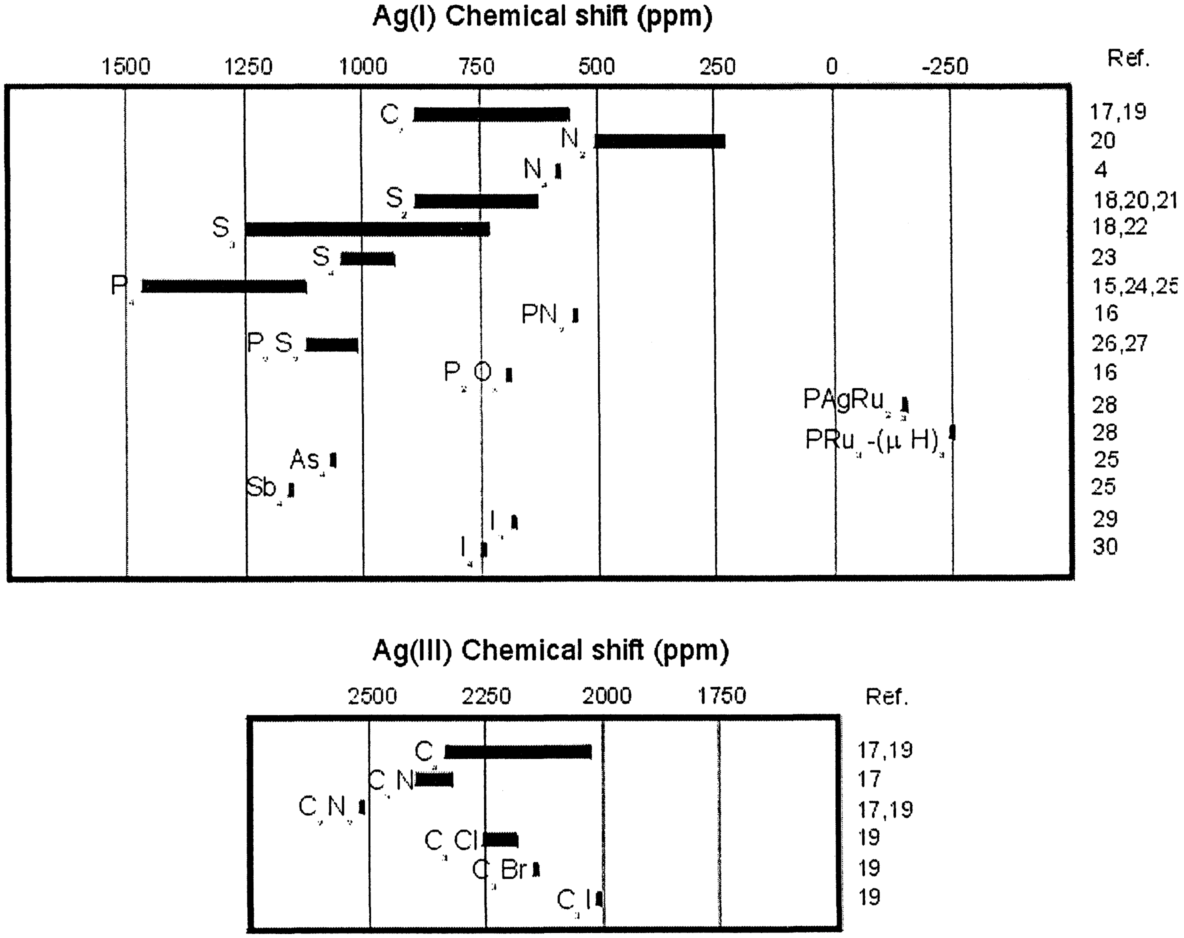

**Figure 3.**

Reported chemical shifts for $^{109}Ag(I)$ and $^{109}Ag(III)$ complexes.

The frequency of a 1M solution of $AgClO_4$ in different nonaqueous solvents ranges from –5 ppm in propylene carbonate to +556ppm in pyridine[31]. Furthermore, due to sometimes labile ligand exchange processes, the absolute chemical shifts need to be interpreted with caution[32]. Frequently, ligand titration studies while monitoring the Ag resonance are used to establish the stoichiometry of certain complexes[32,33].

### SCALAR COUPLING CONSTANTS

To measure the Ag-X coupling constants, it is generally not necessary to acquire $^{109}Ag$ spectra since the splitting can also be extracted from the spectrum of the X-nucleus bound to silver. Due to the limited sensitivity of silver NMR, this alternative has been used quite often. For samples with natural abundance silver in natural abundance, couplings to both $^{109}Ag$ and $^{107}Ag$ are observed. The typical pattern is therefore a doublet of doublets, like the one shown in Figure 4.

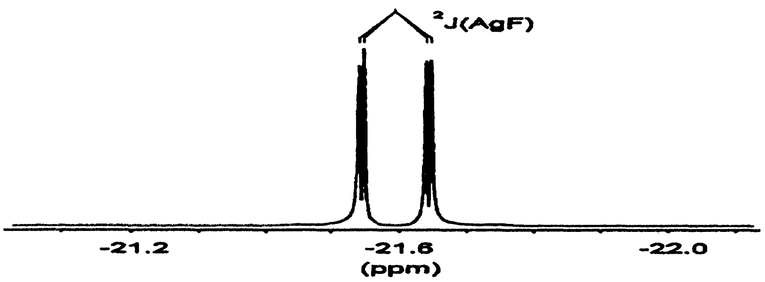

**Figure 4.**

$^{19}F$ spectrum of trans-$[Ag(CF_3)_2(CN)(Cl)]^-$, showing the coupling to both $^{107}Ag$ and $^{109}Ag$.

The coupling constants reported here are always given for the isotope $^{109}Ag$. The corresponding constants for $^{107}Ag$ can be easily calculated from the ratio of the gyromagnetic ratios $\gamma(^{109}Ag)/\gamma(^{107}Ag)=1.15$. A list of reported scalar coupling constants for $^{109}Ag$ is given in Figure 5.

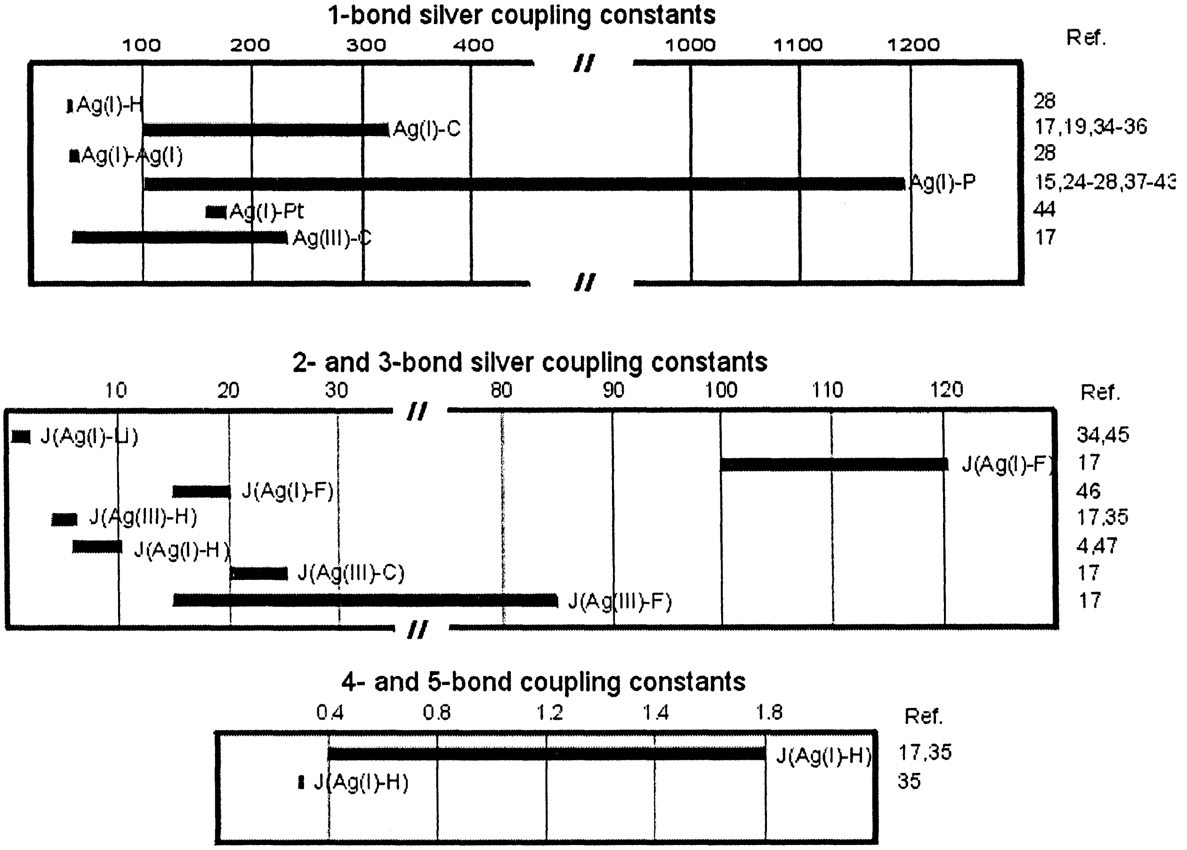

**Figure 5.**
1-5 bond $^{109}Ag$ scalar coupling constants reported in the literature.

The magnitude of the 3-bond silver coupling constant very likely follows a Karplus-type relation, however so far this has not been reported in the literature. In addition to giving structural information, scalar coupling to silver is also essential for the indirect detection of $^{109}Ag$. The scalar silver coupling constant also depends on the coordination number and increases in the series:[47]

$$AgL_4 < AgL_3 < AgL_2 < AgL\ .$$

As an example, the scalar coupling constants are between 177 and 189Hz for $[Ag(CO)_2]^+$, while they are between 245 and 265 for $[AgCO]^+$ compounds[48].

As with the chemical shift scale, the oxidation state is also important for the magnitude of the silver coupling constants, with smaller values for higher oxidation state[17,19].

### CONCLUSIONS

While there are no reported studies of gold NMR, reports of silver NMR studies are growing in the literature. Despite the difficulties associated with NMR spectroscopic studies of silver, chemical shifts and scalar coupling constants have been reported for a variety of Ag complexes. Hopefully, the one demonstration of the use of $^{109}$Ag(I) as an isomorphic, NMR active probe for Cu(I) sites in metalloproteins reported on here will spark additional studies of this nature on other Cu(I) metalloproteins.

### REFERENCES

1. Stocco, F.; Stocco, G.C.; Scovell, W.M.; Tobias, R.S. *Inorg.Chem.* **10**, 2639-2646 (1971); Shaw, C.F.III.; Lundeen, J.W.; Tobias, R.S.; *J.Organomet.Chem.* **51**, 365-374 (1973).
2. Shaw, C.F. III, in The Chemistry of Organic Derivatives of Gold and Silver, Patai, S. and Rappoport, Z. eds., J. Wiley & Sons, Chichester, UK, in press (1999).
3. Heaton, B.T.; Kelsey, R.J. *Inorg.Nucl.Chem.*, **11**, 363-368 (1975).
4. Brevard, C.; van Stein, G.C.; van Koten, G. *J.Am.Chem.Soc.,* **103**, 6746-6748 (1981).
5. Ackermann, J.J.H. *Ph.D. Thesis*, Colorado State University, Fort Collins, CO (1977).
6. Kronenbitter, J. *Ph.D. Thesis*, Eberhard-Karls University of Tübingen, Germany (1977).
7. Kronenbitter, J.; Schweizer, U.; Schwenk, A. *Z.Naturforsch.* **35a**, 319-328 (1980).
8. Brun, E.; Oeser, J.; Staub, H.H.; Telschow, C.G. *Phys.Rev.* **93**, 172-173 (1954).
9. Burges, C.W.; Koschmieder, R.; Sahm, W.; Schwenk, A. *Z.Naturforsch.* **28a**, 1753-1758 (1973).
10. Morris, G.A.; Freeman, R. *J.Am.Chem.Soc.*, **101**, 760-762 (1979).
11. Waugh, J.S. *J.Mol.Spectrosc.* **35**, 298-305 (1970).
12. Doddrell, D.M.; Pegg, D.T.; Bendall, M.R. *J.Magn.Reson.* **48**, 323-327 (1982).
13. Bodenhausen, G.; Ruben, D.J. *Chem.Phys.Lett.* **69**, 185-189 (1980).
14. Müller, L. *J.Am.Chem.Soc.*, **101**, 4481-4484 (1979).
15. Berners-Price, S.J.; Sadler, P.J.; Brevard, C. *Magn.Reson.Chem.* **28**, 145-148 (1990).
16. Lianza, F.; Macchioni, A.; Pregosin, P.; Rüegger, H. *Inorg.Chem.* **33**, 4999-5002 (1994).
17. Eujen, R.; Hoge, B.; Brauer, D.J. *Inorg.Chem.* **36**, 1464-1473 (1997).
18. Narula, S.S.; Mehra, R.K.; Winge, D.R.; Armitage, I.M. *J.Am.Chem.Soc.* **113**, 9354-9358 (1991).
19. Eujen, R.; Hoge, B.; Brauer, D.J. *Inorg.Chem.* **36**, 3160-3166 (1997).
20. Endo, K.; Yamamoto, K.; Matsushita, K.; Deguchi, K.; Kanda, K.; Nakatusji, H *J.Magn.Reson.* **65**, 268-281 (1985).
21. Fijolek, H.G.; González-Durarte, P.; Park, S.H.; Suib, S.L.; Natan, M.J. *Inorg.Chem.*, **36**, 5299-5305 (1997).
22. Narula, S.S.; Winge, D.R.; Armitage, I.M. *Biochemistry*, **32**, 6773-6787 (1993).
23. Fijolek, H.G.; Grohal, J.R.; Sample, J.L.; Natan, M.J. *Inorg.Chem.*, **36**, 622-628 (1997).
24. Black, J.R.; Champness, N.R.; Levason, W.; Reid, G. *J.Chem.Soc.Dalton.Trans.* 3439-3445 (1995).
25. Berners-Price, S.J.; Brevard, C.; Pagelot, A.; Sadler, P.J. *Inorg.Chem.* **24**, 4278-4281 (1985).
26. Hill, A.M.; Levason, W.; Webster, M. *Inorg.Chem.* **35**, 3428-3430 (1996).
27. Doel, C.L.; Gibson, A.M.; Reid, G.; Frampton, C.S. *Polyhedron*, **14**, 3139-3146 (1995).
28. Genge, A.J.R.; Gibson, A.M.; Guymer, N.K.; Reid, G. *J.Chem.Soc.Dalton.Trans.* 4099-4107 (1996).

29. Brown, S.S.D.; Salter, I.D.; Sik, V.; Calquhoun, I.J.; McFarlane, W.; Bates, P.A.; Hursthouse, M.B.; Murray, M. *J.Chem.Soc.Dalton.Trans.* 2177-2185 (1988).
30. Fijolek, H.G.; Oriskovich, T.A.; Benesi, A.J.; González-Durarte, P.; Natan, M.J. *Inorg.Chem.*, **35**, 797-799 (1996).
31. Blenkers, J.; Hofstee, H.K.; Boersma, J.; van der Kerk, G.M. *J.Organomet.Chem.* **168**, 251-258 (1979).
32. Rahimi, A.K.; Popov, A.I. *J.Magn.Reson.* **36**, 351-358 (1979).
33. Nomiya, K.; Tsuda, K.; Kasuga, N.C. *J.Chem.Soc.Dalton.Trans.*, 1653-1659 (1996).
34. Leusink, A.J.; van Koten, G.; Marsman, J.W.; Noltes, J.G. *J.Organomet.Chem.* **55**, 419-425 (1973).
35. Hurlburt, P.K.; Rack, J.J.; Dec, S.F.; Anderson, O.P.; Strauss, S.H. *Inorg.Chem.*, **32**, 373-374 (1993).
36. Yamamoto, Y.; Schmidbaur, H. *J.Organomet.Chem.*, **96**, 133-138 (1975).
37. Socol, S.M.; Verkade, J.G. *Inorg.Chem.*, **28**, 3487-3493 (1984).
38. Socol, S.M.; Jacobson, R.A.; Verkade, J.G. *Inorg.Chem.*, **28**, 88-94 (1984).
39. Bayler, A.; Bowmaker, G.A.; Schmidhaur, H. *Inorg.Chem.*, **35**, 5959-5960 (1996).
40. Adrizzoia, G.A.; La Monica, G.; Maspero, A.; Moret, M.; Masciocchi, N. *Inorg.Chem.*, **36**, 2321-2328 (1997).
41. Lanfredi, A.M.M.; Ugozzoli, F.; Asaro, F.; Pellizer, G.; Marisch, N.; Camus, A. *Inorg.Chim.Acta*, **190**, 71-77 (1991).
42. Bowmaker, G.A.; Effendy, E.; Harvey, P.J.; Healy, P.C.; Skelton, B.W.; White, A.H. *J.Chem.Soc.Dalton.Trans.* 2459-2465 (1996).
43. Bowmaker, G.A.; Effendy, E.; Harvey, P.J.; Healy, P.C.; Skelton, B.W.; White, A.H. *J.Chem.Soc.Dalton.Trans.* 2449-2458 (1996).
44. Baker, L.J.; Bowmaker, G.A.; Camp, D.; Effendy, E.; Healy, P.C.; Schmidbaur, H.; Steigmann, O.; White, A.H. *Inorg.Chem.* **31**, 3656-3662 (1992).
45. Espinet, P.; Foriés, J.; Martínez, F.; Sotes, M.; Lalinde, E.; Teresa-Moreno, M.; Ruiz, A.; Welch, A.J. *J.Organomet.Chem.,* **403**, 253-267 (1991).
46. van der Ploeg, A.F.M.J.; van Koten, G.; Brevard, C. *Inorg.Chem.*, **21**, 2878-2881 (1982).
47. Polishchuk, V.R.; Fedorov, L.A.; Okulevich, P.O.; German, L.S.; Knunyants, I.L. *Tetrahedron.Lett.*, **45**, 3933-3936 (1970).
48. van Stein, G.C.; van der Poel, H.; van Koten, G.; Spek, A.L.; Duisenberg, A.J.M.; Pregosin, P.S. *J.Chem.Soc.Chem.Comm.,* 1016-1018 (1980).

**Received: December 18, 1998 -**
**Accepted in final form: February 19, 1999**

